# Secondary Syphilis

**DOI:** 10.5811/cpcem.2020.7.48515

**Published:** 2020-10-20

**Authors:** Sarah Ehlers, Shane Sergent, John Ashurst

**Affiliations:** Kingman Regional Medical Center, Department of Emergency Medicine, Kingman, Arizona

**Keywords:** Syphilis, secondary syphilis

## Abstract

**Case Presentation:**

A 40-year-old male presented the the emergency department (ED) due to a diffuse body rash after a sexual encounter. Examination revealed a maculopapular rash that included the palms and soles of the feet bilaterally. A rapid plasma reagin was positive, and the patient was treated with 2.4 million units of benzathine benzylpenicillin intramuscularly.

**Diagnosis:**

Secondary syphilis can mimic many disease processes but classically presents as a painless macular rash on the palms of the hands and soles of the feet. Diagnosis is based upon clinical examination coupled with serological testing. Emergency department management should include 2.4 million units of benzathine benzylpenicillin intramuscularly and mitigation strategies.

## CASE PRESENTATION

A 40-year-old male presented to the emergency department due to a diffuse body rash that occurred several weeks earlier. He noted that several weeks prior to the rash developing he was involved in a group sexual encounter and did not use barrier protection. Examination revealed a diffuse maculopapular rash that included the palms and soles of the feet bilaterally (Image). A rapid plasma regain (RPR) was positive, and the patient recalled that he had a painless lesion on the shaft of his penis before the rash developed. The patient was treated with 2.4 million units of benzathine benzylpenicillin intramuscularly and admitted to the medical service for infectious disease consultation.

## DISCUSSION

Over the last several decades there has been a sharp rise in the number of sexually transmitted illnesses across the United States. Syphilis is a genital ulcerative disease caused by the bacterium *Treponema pallidum* and has seen a 72.7% increase in the number of cases since 2013.^1^ Secondary syphilis is the most commonly recognized manifestation of syphilis. The classic rash of secondary syphilis consists of painless, macular, reddish or copper-colored lesions on the palms of the hands or soles of the feet but can be extremely variable.^2^ Lesions can mimic other disease processes including pityriasis rosea, Rocky Mountain spotted fever, contact dermatitis, erythema multiforme, psoriasis, and drug eruptions. Non-cutaneous manifestations can include diffuse lymphadenopathy and hepatosplenomegaly that may mimic mononucleosis or Hodgkin’s lymphoma. Serologic testing with RPR and venereal disease research laboratory tests are most commonly used to diagnosis the disease.^2^ Treatment is 2.4 million units of benzathine benzylpenicillin intramuscularly, which may elicit a Jarisch-Herxheimer reaction.^2^ Patients should be urged to abstain from sexual intercourse and discuss diagnostic strategies and treatment with their sexual partners.

CPC-EM Capsule

What do we already know about this clinical entity?

*There has been a sharp rise in the number of sexually transmitted illnesses annually with syphilis accounting for a 72.7% increase since 2013*.

What is the major impact of the image(s)?

*The classic rash of secondary syphilis are painless, macular, reddish or copper colored lesions on the palms or soles of the feet and is the most commonly recognized form of syphilis*.

How might this improve emergency medicine practice?

*Early recognition, diagnosis, and management can help prevent the spread of sexually transmitted illnesses*.

## Figures and Tables

**Image:**
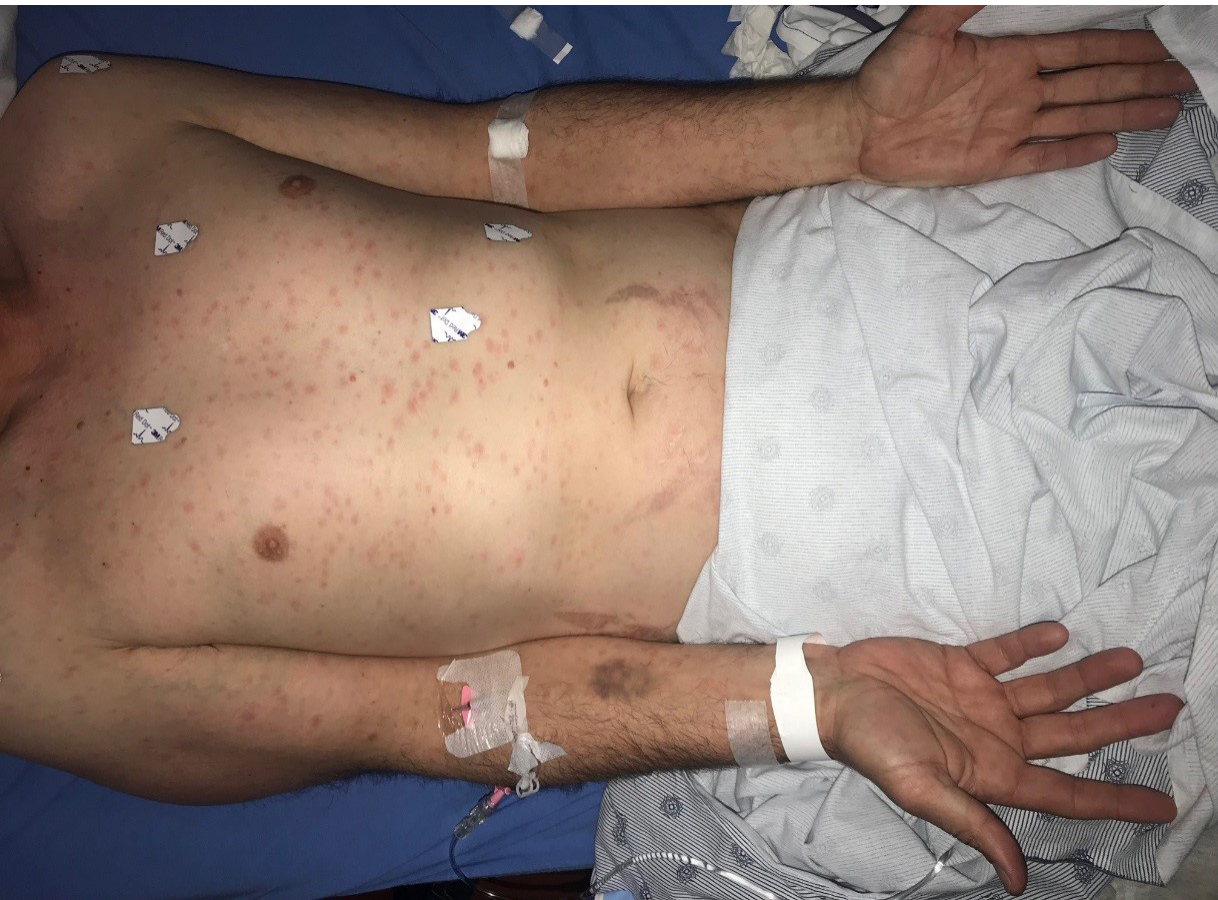
Diffuse maculopapular rash involving the palms bilaterally indicative of secondary syphilis.
